# Dietary vitamin D intake and 2-year changes in cognitive function in older adults with overweight or obesity and metabolic syndrome

**DOI:** 10.1007/s11357-025-01670-1

**Published:** 2025-05-06

**Authors:** Héctor Vázquez-Lorente, Jiaqi Ni, Indira Paz-Graniel, Estefanía Toledo, Dolores Corella, Olga Castañer, J. Alfredo Martínez, Ángel M. Alonso-Gómez, Julia Wärnberg, Jesús Vioque, Dora Romaguera, José López-Miranda, Ramon Estruch, Francisco J. Tinahones, José Lapetra, Lluís Serra-Majem, Amira Bouzalmate-Hajjaj, Josep A. Tur, Rafael M. Micó Pérez, Marta Fanlo, Miguel Delgado-Rodríguez, Ana Barabash Bustelo, Josep Vidal, Clotilde Vázquez, Lidia Daimiel, Emili Ros, Fernando Fernández-Aranda, Teresa Rognoni, Nancy Babio, Eva M. Asensio, Karla-Alejandra Pérez-Vega, Antonio Garcia-Rios, Laura Compañ-Gabucio, Raquel Cueto-Galán, M. Angeles Zulet, Mar Nafria, Rosa Casas, Naomi Cano-Ibáñez, Luis Tojal-Sierra, Ana María Gómez-Pérez, Nuria Goñi, José V. Sorli, María Dolores Zomeño, Antonio P. Arenas-Larriva, Pedro Jiménez-Sellés, Javier Basterra-Gortari, Montserrat Fitó, Jordi Salas-Salvadó

**Affiliations:** 1 Centro de Investigación Biomédica en Red Fisiopatología de La Obesidad y La Nutrición (CIBEROBN), Institute of Health Carlos III, Madrid, Spain; 2 Unitat de Nutrició Humana, Departament de Bioquímica I Biotecnologia, Grup Alimentació, Nutrició, Desenvolupament i Salut Mental ANUT-DSM, Universitat Rovira I Virgili, Reus, Spain; 3 Institut d’Investigació Sanitària Pere Virgili (IISPV), Reus, Spain; 4 Department of Preventive Medicine and Public Health, Instituto de Investigación Sanitaria de Navarra (IdiSNA), University of Navarra, Pamplona, Spain; 5 Department of Nutrition, Harvard T.H. Chan School of Public Health, Boston, MA USA; 6 Department of Preventive Medicine, University of Valencia, Valencia, Spain; 7 CIBER de Epidemiología y Salud Pública (CIBERESP), Instituto de Salud Carlos III (ISCIII), Madrid, Spain; 8 Department of Nutrition, Food Sciences, and Physiology, Center for Nutrition Research, IdiSNA, University of Navarra, Pamplona, Spain; 9 Precision Nutrition and Cardiometabolic Health Program, IEA Food, CEI UAM + CSIC, Madrid, Spain; 10 Departamento de Medicina y Endocrinología, Universidad de Valladolid, Valladolid, Spain; 11 Bioaraba Health Research Institute, Cardiovascular, Respiratory and Metabolic AreaOsakidetza Basque Health Service, Araba University HospitalUniversity of the Basque Country UPV/EHU, Vitoria-Gasteiz, Spain; 12 EpiPHAAN Research Group, School of Health Sciences, University of Málaga – Instituto de Investigación Biomédica en Málaga (IBIMA), Málaga, Spain; 13 Instituto de Investigación Sanitaria y Biomédica de Alicante, Universidad Miguel Hernández (ISABIAL-UMH), Alicante, Spain; 14 Health Research Institute of the Balearic Islands (IdISBa), Palma, Spain; 15 Department of Internal Medicine, Maimonides Biomedical Research Institute of Cordoba (IMIBIC), Reina Sofia University Hospital, University of Cordoba, Cordoba, Spain; 16 Department of Internal Medicine, Institut d’Investigacions Biomèdiques August Pi Sunyer (IDIBAPS), Hospital Clinic, University of Barcelona, Barcelona, Spain; 17 Department of Endocrinology, Virgen de La Victoria Hospital, Instituto de Investigación Biomédica de Málaga (IBIMA), University of Málaga, Málaga, Spain; 18 Department of Family Medicine, Research Unit, Distrito Sanitario Atención Primaria Sevilla, Seville, Spain; 19 Research Institute of Biomedical and Health Sciences (IUIBS), University of Las Palmas de Gran Canaria & Centro Hospitalario Universitario Insular Materno Infantil (CHUIMI), Canarian Health Service, Las Palmas de Gran Canaria, Spain; 20 Department of Preventive Medicine and Public Health, University of Granada, Granada, Spain; 21 Research Group On Community Nutrition & Oxidative Stress, University of Balearic Islands, Palma, Spain; 22 Foundation SEMERGEN (Spanish Society of Primary Care Physicians), Madrid, Spain; 23 Lipids and Vascular Risk Unit, Internal Medicine, Hospital Universitario de Bellvitge-IDIBELL, Hospitalet de Llobregat – Barcelona, Barcelona, Spain; 24 Division of Preventive Medicine, Faculty of Medicine, University of Jaén, Jaén, Spain; 25 Endocrinology and Nutrition Department, Hospital Clínico Universitario San Carlos and Instituto de Investigación Sanitaria del Hospital Clínico San Carlos (IdISSC), Madrid, Spain; 26 Facultad de Medicina, Medicina II Department, Universidad Complutense de Madrid, Madrid, Spain; 27 CIBER Diabetes y Enfermedades Metabólicas (CIBERDEM), Instituto de Salud Carlos III (ISCIII), Madrid, Spain; 28 Department of Endocrinology, Institut d’Investigacions Biomédiques August Pi Sunyer (IDIBAPS), Hospital Clinic, University of Barcelona, Barcelona, Spain; 29 Department of Endocrinology and Nutrition, Hospital Fundación Jimenez Díaz, Instituto de Investigaciones Biomédicas IISFJD, University Autonoma, Madrid, Spain; 30 Nutritional Control of the Epigenome Group, Precision Nutrition and Obesity Program, IMDEA Food, CEI UAM + CSIC, Madrid, Spain; 31 Departamento de Ciencias Farmacéuticas y de La Salud, Faculty de Farmacia, Universidad San Pablo-CEU, CEU Universities, Boadilla del Monte, Spain; 32 Lipid Clinic, Department of Endocrinology and Nutrition, Institut d’Investigacions Biomèdiques August Pi Sunyer (IDIBAPS), Hospital Clínic, Barcelona, Spain; 33 Psychoneurobiology of Eating and Addictive Behaviors Group, Institut d’Investigació Biomèdica de Bellvitge (IDIBELL), Barcelona, Spain; 34 Department of Clinical Psychology, University Hospital of Bellvitge and University of Barcelona, Barcelona, Spain; 35 Department of Neurology, Clínica Universidad de Navarra, Madrid, Spain; 36 Unit of Cardiovascular Risk and Nutrition, Hospital del Mar Research Institute (IMIM), Barcelona, Spain; 37 Department of Public Health and Psychiatry, School of Medicine, University of Malaga, Malaga, Spain; 38 Biomedical Research Institute of Malaga (IBIMA), Malaga, Spain; 39 Instituto de Investigación Biosanitaria de Granada (IBS-Granada), Granada, Spain; 40 Navarra Health Service (Osasunbidea), Primary Care, Pamplona, Navarra Spain; 41 Primary Health Care Center Santa Pola, Alicante, Spain; 42 Institut de Recerca en Nutrició I Seguretat Alimentaria (INSA-UB), University of Barcelona, Barcelona, Spain

**Keywords:** Vitamin D, Cognition, Cognitive decline, Cognitive function, Aging, Older people

## Abstract

The protective role of dietary vitamin D intake on cognitive function is of interest, but evidence remains inconsistent. We aimed to evaluate the association between dietary vitamin D intake and 2-year cognitive changes in older adults at risk of cognitive decline. This longitudinal study comprised 5454 individuals (aged 55–75 years in men and 60–75 years in women) who exhibited overweight/obesity and metabolic syndrome. Data on dietary vitamin D intake was assessed using a validated 143-item food frequency questionnaire. Cognitive function was assessed through five composite scores derived from a comprehensive battery of eight neuropsychological tests, encompassing global cognitive function, general cognitive function, attention, executive function, and language domains. Multivariable-adjusted linear regression models were fitted to examine the association between energy-adjusted cumulative average dietary vitamin D intake over time and 2-year changes in cognitive function. After adjusting for multiple covariates, energy-adjusted cumulative average dietary vitamin D intake as a continuous variable was associated with greater 2-year improvements in global cognitive function (*β* 1.18 × 10^−2^; 95% CI 0.19 × 10^−2^ to 2.17 × 10^−2^), executive function (*β* 1.12 × 10^−2^; 95% CI 0.03 × 10^−2^ to 2.21 × 10^−2^), and language (*β* 1.61 × 10^−2^; 95% CI 0.43 × 10^−2^ to 2.78 × 10^−2^). Additionally, the higher cumulative average dietary vitamin D intake quartile was associated with an increase in global cognitive function (*β* 7.10 × 10^−2^; 95% CI 0.59 × 10^−2^ to 13.6 × 10^−2^), language (*β* 7.07 × 10^−2^; 95% CI − 0.52 × 10^−2^ to 14.7 × 10^−2^), and a lower decline in attention (*β* 9.58 × 10^−2^; 95% CI 1.60 × 10^−2^ to 17.5 × 10^−2^). A higher dietary vitamin D intake was associated with modest favorable changes in cognitive function and a reduced cognitive decline over a 2-year period. These findings highlight the need for further research to explore the potential benefits of boosting dietary vitamin D intake for cognitive health in older adults.

**Supplementary Information:**

The online version contains supplementary material available at 10.1007/s11357-025-01670-1.

## Introduction

Cognitive impairment and dementia have become major healthcare and public health concerns [1]. Projections indicate a substantial increase by 2050 in developed countries [2], primarily due to obesity, aging, and unhealthy lifestyles [3]. Effective prevention and control measures are urgently required [4]. Identifying strategies to prevent these conditions by focusing on modifiable risk factors, such as lifestyle behaviors, specifically on diet, could potentially mitigate or delay their onset [5].

Vitamin D metabolites, along with the enzymes involved in its metabolism and its receptors, have been identified in brain regions involved in cognitive processes [6]. Acting as a neurosteroid, vitamin D is involved in behavioral function, neurogenesis, and neuroprotection [7]. It has been reported that individuals with low blood vitamin D concentrations present poorer cognitive function and a higher risk of cognitive decline compared to those with an adequate status [8]. Indeed, individuals with vitamin D deficiency have been shown to have twice the risk of all-cause dementia [9]. Vitamin D is acquired through cutaneous synthesis from sun exposure and dietary intake. Although dietary intake has traditionally been considered a minor source, it becomes crucial for older adults and those with excess weight, as they derive significantly less vitamin D from cutaneous synthesis [10, 11].

Emerging evidence indicates that dietary intake of vitamin D, rather than from supplements, may have a protective association with cognitive function [12]. Inadequate dietary vitamin D intake has been associated with poorer cognitive function and up to 30% increased risk of cognitive impairment [13]. However, epidemiological evidence on this topic remains limited. Cross-sectional studies have suggested a beneficial link between dietary vitamin D intake and cognitive function [14–17], but findings from longitudinal studies have been mixed, showing either slight and modest benefits [18–20] or no clear associations with cognitive function over time [21]. In addition, the impact of vitamin D supplementation on cognition remains unclear, as most intervention studies have not demonstrated significant benefit [22]. Discrepancies among research studies may stem from differences in cognitive assessment tools, supplementation dosage, and duration of the intervention, as well as baseline characteristics of study participants, especially in the initial vitamin D status [23–25].

Therefore, the evidence regarding the beneficial effects of dietary vitamin D intake on cognitive function and/or cognitive decline remains inconclusive. Unraveling a possible connection between dietary vitamin D intake and cognitive function is important for considering healthcare initiatives to prevent or delay cognitive decline [26], particularly among aged and obese individuals, whose vitamin D levels and cognitive health tend to be reduced [27, 28]. Therefore, the aim of this study is to evaluate the association between dietary vitamin D intake and 2-year cognitive changes in older adults with overweight/obesity and metabolic syndrome, who are at risk of cognitive decline. We hypothesize that a higher dietary vitamin D intake may be associated with better cognitive function compared to lower intake over a 2-year follow-up.

## Materials and methods

### Study design

This study was performed within the framework of the PREDIMED-Plus trial. The trial, ongoing in Spain across 23 centers, aims to assess the impact of an energy-reduced Mediterranean diet (MedDiet), increased physical activity, and behavioral modification on cardiovascular health [29]. More details on the trial protocol are available at https://www.predimedplus.com/ and in published sources [30, 31]. Ethical approval was obtained from all participating centers, and written informed consent was obtained from all participants. The trial was registered in 2014 at the International Standard Randomized Controlled Trial registry [ISRCT; www.isrctn.com/ISRCTN89898870].

### Participants

**Fig. 1:**
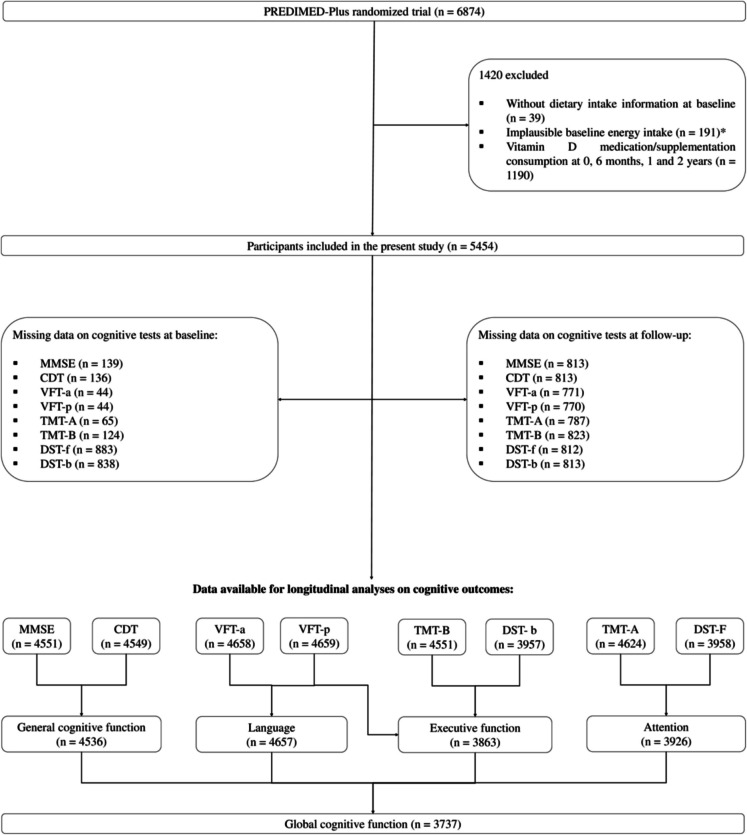
Flowchart of the study population. CDT, Clock Drawing test; DST-b, Digit Span test backward; DST-f, Digit Span test forward; MMSE, Mini-Mental State Examination; TMT-A, Trail Making test part A; TMT-B, Trail Making test part B; VFT-a, verbal fluency tasks semantical; VFT-p, verbal fluency tasks phonological. Asterisk (*) symbols indicate daily energy intakes for men < 800 kcal or > 4000 kcal and women < 500 kcal or > 3500 kcal

The study enrolled community-dwelling adults aged 55 to 75 years with overweight or obesity (body mass index (BMI) ranging from 27 to 40 kg/m^2^) and at least three criteria for metabolic syndrome, as defined by established criteria [32]. Participants enrolled in this study had preserved cognitive function to understand and give consent and were not institutionalized. From October 2013 to December 2016, a total of 6874 eligible participants were randomly assigned in a 1:1 ratio to either the intervention group or the control group receiving usual care (traditional energy-unrestricted MedDiet). Randomization was performed centrally using a computer-generated system, with stratification by center, sex, and age. Couples sharing a household were randomized together. The randomization process was blinded to staff and investigators. For the purpose of the present study, participants not completing dietary questionnaires, reporting energy intakes outside predefined limits (< 800 to ≥ 4000 kcal/day for men, < 500 to ≥ 3500 kcal/day for women) at baseline, or taking vitamin D medication/supplementation at baseline, 6 months, 1 year, and 2 years of follow-up were excluded [33]. A total of 5454 participants were finally included in the study (Fig. 1).

### Exposure: vitamin D intake

Trained dietitians conducted face-to-face interviews with participants to assess their dietary habits using a validated 143-item food frequency questionnaire (FFQ) [34]. The FFQ comprised nine response options ranging from “never” to “more than six times per day.” These responses were converted into daily intake values using standard portion sizes for each item. The Spanish food composition database was utilized to estimate the energy and dietary vitamin D intake [35]. Dietary vitamin D intake was assessed at baseline, 6 months, 1 year, and 2 years of follow-up. The energy-adjusted cumulative average of dietary vitamin D intake over these time points was subsequently calculated by summing the reported dietary vitamin D intake from each time point and dividing by the number of assessments available up to that moment, and using the residual method [36] to better represent long-term intake and reduce within-person variation and measurement errors. Participants were additionally categorized into quartiles of energy-adjusted cumulative average dietary vitamin D intake over time.

### Outcome: changes in cognitive function

Cognitive function evaluation was conducted by trained personnel both at baseline and at the 2-year follow-up assessment. A battery of eight neuropsychological tests, validated specifically for the Spanish population, was administered via individual interviews. These tests comprised the Mini-Mental State Examination (MMSE), the Clock Drawing test (CDT), the Verbal Fluency tests (VFTs), the forward and backward versions of the Digit Span test (DST-f and DST-b, respectively) from the Wechsler Adult Intelligence Scale-III (WAIS-III), and the Trail Making test parts A (TMT-A) and B (TMT-B). Detailed descriptions of these neuropsychological assessments can be found elsewhere [37]. Each cognitive test administered at baseline and during the 2-year follow-up period was standardized for every participant to a *z*-score, utilizing the mean and standard deviation (SD) derived from the baseline data. Subsequently, the difference between the standardized scores at the two time points was calculated to assess changes in cognitive function over time [38, 39].

Composite measures for four cognitive composite scores (general cognitive function, executive function, attention, and language), as well as a global assessment of cognitive function (GCF), were computed for each participant. The composite cognitive assessments were determined by aggregating or deducting individual test *z*-scores based on whether a higher score indicates superior or inferior cognitive function, respectively, as detailed in Table S1. Following this procedure, the resulting five composite scores were further standardized to *z*-scores using the mean and standard deviation (SD) values from baseline composite score data. The changes observed in these five composite scores over the 2-year period constituted the primary outcome of interest in this study.

### Covariate assessments

Sociodemographic and lifestyle information encompassing age, sex, education level, civil status, and smoking habits were gathered by administered questionnaires. Physical activity was estimated utilizing a validated Spanish short version of the Minnesota Leisure Time Physical Activity Questionnaire (the REGICOR questionnaire) [40]. Personal medical history, encompassing conditions such as type 2 diabetes, hypertension, and hypercholesterolemia, and medication usage were either self-reported or extracted from medical records. Depressive symptomatology was evaluated using the Beck Depression Inventory-II (BDI-II), where depression risk was established as a score ≥ 14 [41, 42]. Anthropometric variables, such as weight and height, were assessed using calibrated scales and wall-mounted stadiometers, respectively. BMI was calculated as weight (kg)/height (m)^2^. Food group information, including variables related to vegetables, fruits, legumes, cereals, meat, fish, dairy, nuts, oils and fats, olive oils, biscuits, coffee, tea, and alcohol consumption, was collected using the same FFQ used to assess dietary vitamin D intake. Subsequently, energy and nutrient intake estimations were derived utilizing the Spanish Food Composition Tables [35]. Energy-adjusted cumulative averages for dietary variables were obtained using the residual method [36].

### Statistical analyses

Baseline characteristics of the study cohort were presented both in overall and categorized by quartiles of energy-adjusted cumulative average dietary vitamin D intake over time as means ± SDs for continuous variables and numbers (percentages) for categorical variables. One-way analysis of variance (ANOVA) was employed for continuous variables, while chi-square tests were utilized for categorical variables.

The associations between energy-adjusted cumulative average dietary vitamin D intake (exposure) and changes observed in cognitive function measurements over a 2-year period (outcome) were investigated through multivariable linear regression models. These models were adjusted for potential confounders pertinent to cognitive function and were presented as *β*-coefficients along with their corresponding 95% confidence intervals (CIs).

A basic model was adjusted for respective baseline cognitive function scores, age (years), and sex (men/women). The intervention group (control or intervention), geographic area of the participating centers (south/north), education level (primary or less, secondary, college), civil status (single, divorced or separated, married, widower), BMI (kg/m^2^), physical activity (METs/min/day), smoking status (never, former, current), energy-adjusted cumulative average alcohol consumption in g/day (and adding the quadratic term), baseline presence of depressive symptomatology (yes/no), type 2 diabetes (yes/no), hypertension (yes/no), hypercholesterolemia (yes/no), and energy-adjusted cumulative average consumption of food groups (i.e., vegetables, fruits, legumes, cereals, dairy, meat, fish, nuts, oils and fats, olive oils, biscuits (in g/day), coffee, and tea (in mL/day)) were additionally included as covariates in the multivariable model. Penalized splines were used to explore the potential nonlinearity association using the continuous variable of cumulative average dietary vitamin D intake [43].

Robust variance estimators were utilized in all models to accommodate intra-cluster correlations, as couples from the same household were randomized together. To explore potential effect modification by age, sex, intervention group, educational level, BMI, smoking, type 2 diabetes status, and physical activity, we used the likelihood ratio test by comparing models with and without the multiplicative interaction term between these factors and energy-adjusted cumulative average dietary vitamin D intake within fully adjusted models. Sensitivity analyses were conducted to assess the robustness of the results, including the exclusion of individuals who met the following criteria at baseline: (1) MMSE < 24 and (2) extreme percentiles of GCF *z*-scores (< 2.5%, > 97.5%).

All statistical analyses were conducted with Stata/SE version 14.2 (StataCorp LLC, College Station, TX, USA) using the PREDIMED-Plus study dataset updated to December 19, 2023. All graphs were plotted using GraphPad Prism software v.9.0 (GraphPad Software, San Diego, CA, USA). Statistical significance was defined as a two-tailed *P*-value < 0.05.

## Results

Table 1 presents the baseline characteristics of the overall study population and by quartiles of energy-adjusted cumulative average dietary vitamin D intake over time. The energy-adjusted cumulative average daily dietary vitamin D intake ranged from 3.4 ± 1.5 µg/day in the lowest quartile to 9.3 ± 3.3 µg/day in the highest quartile (overall mean: 6.0 ± 3.4 µg/day). Participants in the highest quartile had a lower prevalence of hypertension, tended to take fewer antidiabetic drugs, had a higher educational level, and engaged in more physical activity. Additionally, they exhibited higher baseline *z*-scores in global cognitive function, general cognitive function, executive function, and language (all *P* ≤ 0.047; Table 1). In terms of dietary intake, participants in the highest quartile of energy-adjusted cumulative average dietary vitamin D intake consumed lower quantities of carbohydrates and higher amounts of protein, fat, fiber, vegetables, fruits, legumes, cereals, fish, nuts, oils and fats, olive oils, and alcohol compared to those in the lowest quartile (all *P* ≤ 0.014; Table S2). Over a 2-year follow-up period, improvements in global cognitive function, general cognitive function, executive function, and language were observed, while a decline in the attention domain was noted over this time period across categories of dietary vitamin D intake (Table S3). However, this decline in attention was less pronounced in participants with the highest average dietary vitamin D intake (Table S3).

**Table 1:** Baseline characteristics of the PREDIMED-Plus participants both in overall and by quartiles of energy-adjusted cumulative averages of dietary vitamin D intake

	Total population	Categories of dietary vitamin D intake				*P*-value^1^
		1 st quartile	2nd quartile	3rd quartile	4 th quartile	
Vitamin D (µg/day)	6.0 ± 3.4	3.4 ± 1.5	5.0 ± 2.2	6.5 ± 3.0	9.3 ± 3.3	**0.001**
Sociodemographic variables						
Age (years)	64.8 ± 4.9	64.6 ± 5.1	64.6 ± 5.0	64.9 ± 4.9	64.9 ± 4.8	0.279
Women (*n*/%)	2285 (41.9)	492 (36.1)	565 (41.5)	593 (43.5)	635 (46.6)	**0.001**
Education level (*n*/%)						
Primary or less	2627 (48.2)	692 (50.7)	682 (50.0)	673 (49.3)	580 (42.6)	
Secondary	1613 (29.6)	377 (27.6)	411 (30.2)	404 (29.6)	421 (30.9)	**0.001**
College	1214 (22.3)	295 (21.6)	270 (19.8)	287 (21.0)	362 (26.6)	
Civil status (*n*/%)						
Single, divorced, or separated	670 (12.3)	171 (12.5)	166 (12.2)	147 (10.8)	186 (13.6)	
Married	4258 (78.0)	1066 (78.2)	1071 (78.6)	1065 (78.1)	1056 (77.5)	0.164
Widower	526 (9.6)	127 (9.3)	126 (9.2)	152 (11.1)	121 (8.9)	
Disease presence or medication usage at recruitment						
Type 2 diabetes (*n*/%)	1746 (32.0)	441 (32.3)	457 (33.5)	446 (32.7)	402 (29.5)	0.123
Hypertension (*n*/%)	4576 (83.9)	1134 (83.1)	1160 (85.1)	1165 (85.4)	1117 (82.0)	**0.042**
Hypercholesterolemia (*n*/%)	3821 (70.1)	940 (68.9)	980 (71.9)	955 (70.0)	946 (69.4)	0.343
Depressive symptomatology, (*n*/%)	1039 (19.1)	260 (19.1)	263 (19.3)	259 (19.0)	257 (18.9)	0.993
Medication use (*n*/%)						
Insulin or other antidiabetic drugs	1487 (27.3)	380 (27.9)	390 (28.6)	385 (28.2)	332 (24.4)	**0.047**
Antihypertensive agents	4269 (78.3)	1062 (77.9)	1079 (79.2)	1078 (79.0)	1050 (77.0)	0.482
Statins or other hypolipidemic drugs	2818 (51.7)	687 (50.4)	730 (53.6)	689 (50.5)	712 (52.2)	0.286
Anthropometric variables						
BMI (kg/m^2^)	32.5 ± 3.4	32.6 ± 3.4	32.5 ± 3.5	32.5 ± 3.4	32.3 ± 3.5	0.231
Waist circumference (cm)						
Women	103.6 ± 9.1	103.8 ± 8.6	103.3 ± 9.3	104.0 ± 9.4	103.5 ± 9.1	0.542
Men	110.7 ± 8.7	110.9 ± 9.0	110.9 ± 8.8	110.7 ± 8.5	110.4 ± 8.5	0.631
Lifestyle variables						
Physical exercise (METs/min/day)	364.3 ± 335.0	344.3 ± 335.2	346.2 ± 316.4	363.7 ± 338.8	403.0 ± 346.0	**0.001**
Smoking status, (*n*/%)						
Current smoker	717 (13.2)	222 (16.3)	182 (13.4)	152 (11.1)	161 (11.8)	**0.001**
Former smoker	2452 (45.0)	610 (44.7)	621 (45.6)	600 (44.0)	621 (45.6)	
Never smoker	2285 (41.9)	532 (39.0)	560 (41.1)	612 (44.9)	581 (42.6)	
Cognitive function assessment						
Global cognitive function (*n* = 3737)^2^	4.1 ± 1.0	2.1 ± 1.0	6.0 ± 1.0	2.6 ± 1.0	11.8 ± 1.0	**0.001**
General cognitive function (*n* = 4536)^2^	− 0.8 ± 1.0	0.6 ± 1.0	− 5.4 ± 1.0	− 1.8 ± 1.0	6.6 ± 1.0	**0.033**
Executive function (*n* = 3863)^2^	− 0.9 ± 1.0	− 4.2 ± 1.0	− 4.1 ± 1.0	− 4.2 ± 1.0	12.6 ± 1.0	**0.001**
Attention (*n* = 3926)^2^	− 0.3 ± 1.0	− 0.5 ± 1.0	− 5.6 ± 1.0	0.0 ± 1.0	6.1 ± 1.0	0.080
Language (*n* = 4657)^2^	− 0.7 ± 1.0	− 3.5 ± 1.0	− 2.5 ± 1.0	− 7.1 ± 1.0	13.1 ± 1.0	**0.001**

Data are presented as *n* (%) or mean ± SD for categorical and continuous variables, respectively. Significant values (*P* < 0.05) were highlighted in bold type

*BMI* body mass index, *CI* confidence interval, *METs* metabolic equivalents

^1^The *p*-value for differences between categories of energy-adjusted cumulative average dietary vitamin D intake was calculated by Pearson’s chi-square test or one-way ANOVA, as appropriate

^2^Values are presented as multiples of 10^−2^ (× 10^−2^)

Table 2 displays the longitudinal associations (*β* coefficients and 95% CI) between energy-adjusted cumulative average dietary vitamin D intake and changes in cognitive function over the 2-year follow-up period. Results from multivariable adjusted models show significant positive associations between energy-adjusted cumulative average dietary vitamin D intake and 2-year beneficial changes in cognitive function. In particular, 1 µg/day higher energy-adjusted cumulative average dietary vitamin D intake was associated with more beneficial changes in global cognitive function (*β* 1.18 × 10^−2^; 95% CI 0.19 × 10^−2^ to 2.17 × 10^−2^; *P* 0.019), executive function (*β* 1.12 × 10^−2^; 95% CI 0.03 × 10^−2^ to 2.21 × 10^−2^; *P* 0.044), and language (*β* 1.61 × 10^−2^; 95% CI 0.43 × 10^−2^ to 2.78 × 10^−2^; *P* 0.007). Results from the potential nonlinearity exploration did not show any clear departure from the linearity of the associations (data not shown). Additionally, participants in the highest quartile of energy-adjusted cumulative average dietary vitamin D intake showed better cognitive function evolution after 2 years of follow-up than those in the lowest quartile, specifically, in global cognitive function (*β* 7.10 × 10^−2^; 95% CI 0.59 × 10^−2^ to 13.6 × 10^−2^; *P*-trend 0.025), attention (*β* 9.58 × 10^−2^; 95% CI 1.60 × 10^−2^ to 17.5 × 10^−2^; *P*-trend 0.039), and language (*β* 7.07 × 10^−2^; 95% CI − 0.52 × 10^−2^ to 14.7 × 10^−2^; *P*-trend 0.039). However, no significant association between energy-adjusted cumulative average dietary vitamin D intake and general cognitive function was shown.

**Table 2:** Longitudinal association between energy-adjusted cumulative average dietary vitamin D intake and changes in cognitive function over 2 years of follow-up in the PREDIMED-Plus cohort

	Continuous		Categories of dietary vitamin D intake				*P*-trend
	Dietary vitamin D intake (µg/day)		1 st quartile	2nd quartile	3rd quartile	4 th quartile	
	*β* [95% CI]^1^	*P*-value	*β* [95% CI]^1^	*β* [95% CI]^1^	*β* [95% CI]^1^	*β* [95% CI]^1^	
Global cognitive function (*n*)	(*n* = 3737)	**–**	(*n* = 935)	(*n* = 934)	(*n* = 934)	(*n* = 934)	**–**
Mean ± SD dietary vitamin D intake	6.54 ± 2.42	**–**	3.61 ± 0.82	5.48 ± 0.50	7.28 ± 0.56	9.79 ± 1.21	**–**
Basic model	**1.08 [0.39, 1.58]**	**0.002**	Reference	1.49 [− 3.52, 6.49]	3.94 [− 1.03, 8.91]	**6.88 [2.03, 11.7]**	**0.003**
Multivariable-adjusted model	**1.18 [0.19, 2.17]**	**0.019**	Reference	1.74 [− 3.37, 6.86]	4.47 [− 1.13, 10.1]	**7.10 [0.59, 13.6]**	**0.025**
General cognitive function (*n*)	(*n* = 4536)	**–**	(*n* = 1134)	(*n* = 1134)	(*n* = 1134)	(*n* = 1134)	**–**
Mean ± SD dietary vitamin D intake	6.56 ± 2.42	**–**	3.63 ± 0.82	5.51 ± 0.49	7.31 ± 0.64	9.81 ± 1.23	**–**
Basic model	0.30 [− 0.65, 1.26]	0.534	Reference	− 0.91 [− 7.65, 5.83]	− 1.30 [− 7.90, 5.30]	1.89 [− 4.72, 8.50]	0.579
Multivariable-adjusted model	− 0.17 [− 1.55, 1.20]	0.805	Reference	− 2.00 [− 8.98, 4.97]	− 2.65 [− 10.0, 4.75]	− 1.69 [− 10.5, 0.71]	0.722
Executive function (*n*)	(*n* = 3863)	**–**	(*n* = 966)	(*n* = 966)	(*n* = 966)	(*n* = 965)	**–**
Mean ± SD dietary vitamin D intake	6.55 ± 2.42	**–**	3.63 ± 0.82	5.50 ± 0.49	7.28 ± 0.56	9.79 ± 1.21	**–**
Basic model	**1.42 [0.67, 2.15]**	**0.001**	Reference	− 1.17 [− 6.36, 4.02]	4.27 [− 0.93, 9.47]	**7.67 [2.54, 12.8]**	**0.001**
Multivariable-adjusted model	**1.12 [0.03, 2.21]**	**0.044**	Reference	− 1.43 [− 6.71, 3.85]	3.10 [− 2.83, 9.05]	4.98 [− 2.00, 12.0]	0.084
Attention (*n*)	(*n* = 3926)	**–**	(*n* = 982)	(*n* = 981)	(*n* = 982)	(*n* = 981)	**–**
Mean ± SD dietary vitamin D intake	6.56 ± 2.43	**–**	3.63 ± 0.82	5.51 ± 0.49	7.30 ± 0.56	9.81 ± 1.22	**–**
Basic model	**1.28 [0.39, 2.16]**	**0.005**	Reference	5.70 [− 0.39, 11.8]	3.13 [− 3.34, 9.59]	**10.5 [− 3.33, 16.6]**	**0.003**
Multivariable-adjusted model	1.12 [0.10, 2.35]	0.073	Reference	5.26 [− 0.93, 11.4]	3.56 [− 3.60, 10.7]	**9.58 [1.60, 17.5]**	**0.039**
Language (*n*)	(*n* = 4657)	**–**	(*n* = 1165)	(*n* = 1164)	(*n* = 1164)	(*n* = 1164)	**–**
Mean ± SD dietary vitamin D intake	6.58 ± 2.43	**–**	3.64 ± 0.83	5.53 ± 0.49	7.31 ± 0.56	9.82 ± 1.23	**–**
Basic model	**2.12 [1.31, 2.93]**	**0.001**	Reference	1.70 [− 4.01, 7.41]	7.60 [19.7, 13.2]	**12.1 [6.57, 17.8]**	**0.001**
Multivariable-adjusted model	**1.61 [0.43, 2.78]**	**0.007**	Reference	0.54 [− 5.27, 6.36]	4.95 [− 1.42, 11.3]	**7.07 [− 0.52, 14.7]**	**0.039**

Basic models were adjusted for the respective cognitive test score at baseline, age (years), and sex. Multivariable-adjusted models were further adjusted for the intervention group, geographic area of the participating centers (south/north), education level (primary, secondary, or college), civil status (single, divorced or separated, married, widower), body mass index (kg/m^2^), physical activity (METs/min/day), smoking status (current, former, or never), energy-adjusted cumulative average of alcohol consumption in g/day (and adding the quadratic term), depressive symptomatology (yes/no), diabetes prevalence (yes/no), hypertension prevalence (yes/no), hypercholesterolemia prevalence (yes/no), and energy-adjusted cumulative average consumption of food groups (vegetables, fruits, legumes, cereals, oils and fats, olive oils, biscuits, meat, fish, dairy, nuts [g/day], coffee and tea [mL/day]). *β*-Coefficients were estimated using linear regression models with robust standard errors to account for intracluster correlations. Linear trend was calculated by assigning the median values to each quartile of energy-adjusted cumulative average dietary vitamin D intake and treating these values across groups as a continuous variable in the linear regression models. Significant values (*P* < 0.05) were highlighted in bold type

*CI* confidence interval

^1^*β* [95% CI] values are expressed as multiples of 10^−2^ (× 10^−2^)

**Fig. 2:**
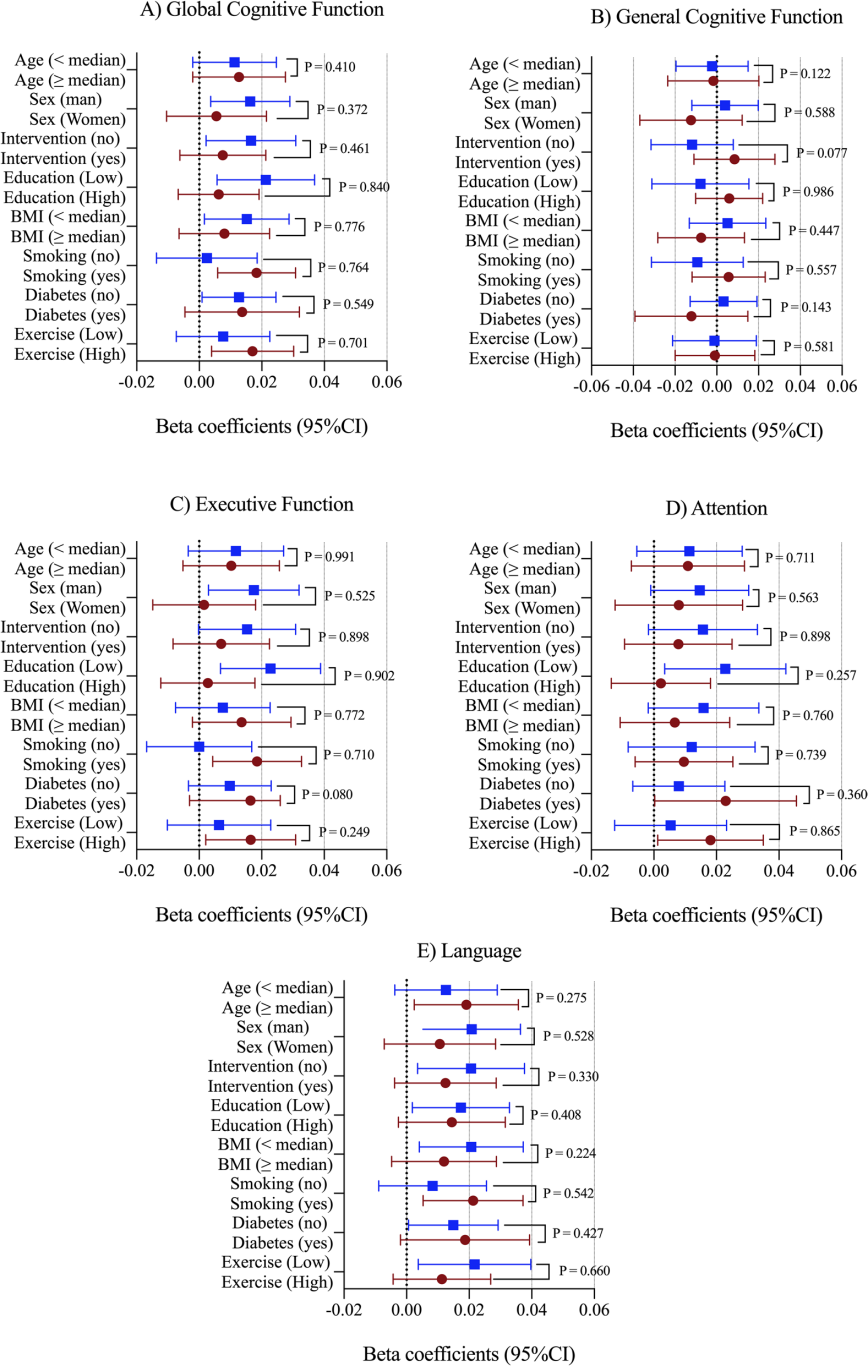
Interaction between energy-adjusted cumulative average dietary vitamin D intake and different baseline variables of the study potentially related to cognitive function. Abbreviations: BMI, body mass index; CI, confidence interval. Multivariable-adjusted models were adjusted for the respective cognitive test score at baseline, age (years), sex, intervention PREDIMED-Plus randomized groups, and participating center (south/north), education level (primary, secondary, or college), civil status (single, divorced or separated, married, widower), body mass index (kg/m^2^), physical activity (METs/min/day), smoking status (current, former, or never), energy-adjusted cumulative average alcohol consumption in g/day (and adding the quadratic term), depressive symptomatology (yes/no), diabetes prevalence (yes/no), hypertension prevalence (yes/no), hypercholesterolemia prevalence (yes/no), and energy-adjusted cumulative average consumption of food groups (vegetables, fruits, legumes, cereals, oils and fats, olive oils, biscuits, meat, fish, dairy, nuts [g/day], coffee and tea [mL/day]). Significant values (*P* < 0.05) were highlighted in bold type

Figure 2 illustrates the results of the interaction analyses between energy-adjusted cumulative average dietary vitamin D intake and various baseline variables potentially related to cognitive function. No significant interactions were found between energy-adjusted cumulative average dietary vitamin D intake and categories of age, sex, intervention group, educational level, BMI, smoking status, type 2 diabetes, and physical activity across all assessed cognitive function domains (all *P* ≥ 0.080; Fig. 2A–E).

**Fig. 3:**
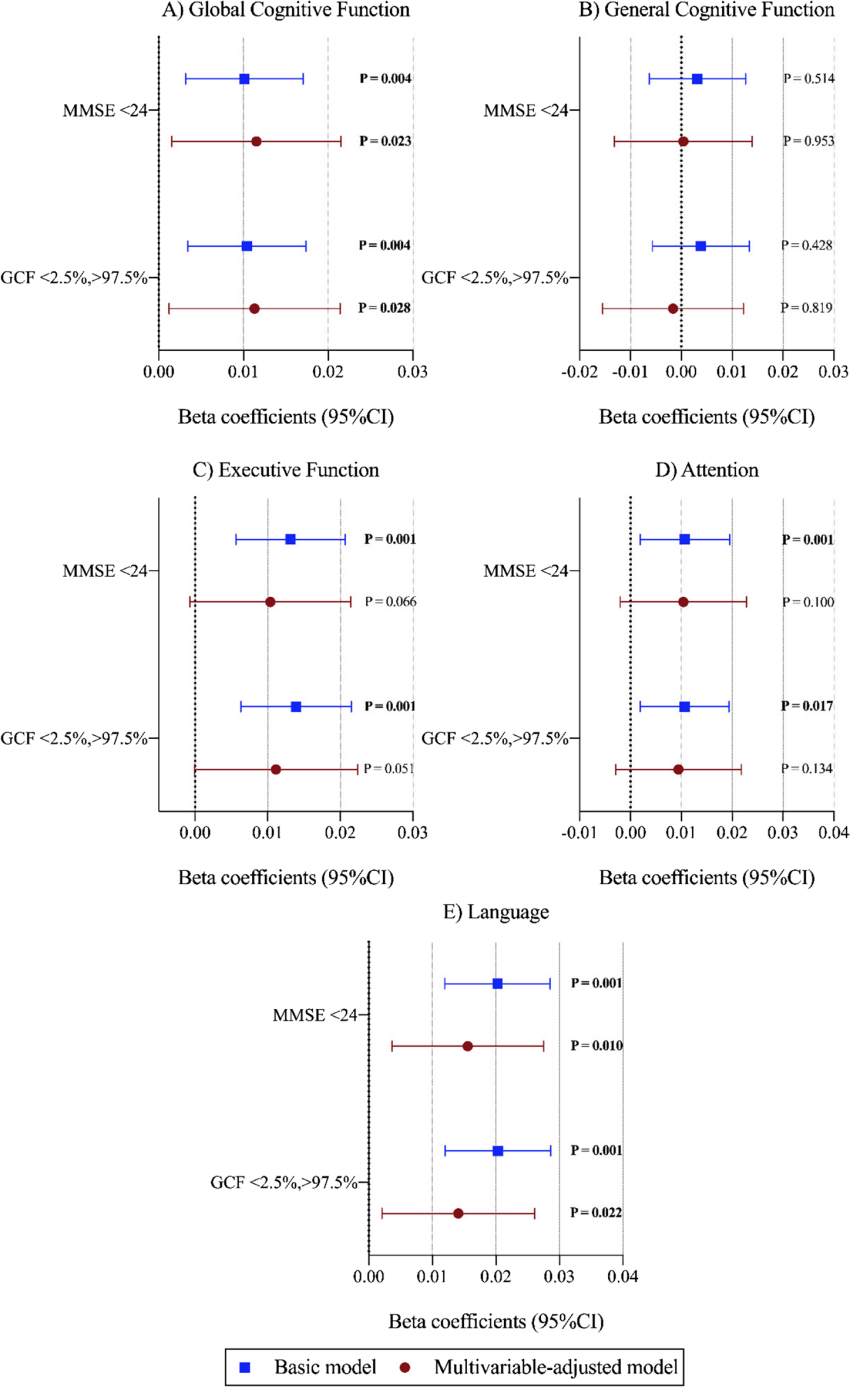
Sensitivity analyses for the longitudinal association between energy-adjusted cumulative average dietary vitamin D intake and changes in cognitive function over a 2-year follow-up period in the PREDIMED-Plus cohort. Abbreviations: GCF, global cognitive function; MMSE, Mini-Mental State Examination. From the top to the bottom, removal of participants at baseline (1) with MMSE < 24, (2) with extreme percentiles of GCF *z*-score (< 2.5%, > 97.5%). Basic models were adjusted for the respective cognitive test score at baseline, age (years), and sex. Multivariable-adjusted models were adjusted for the respective cognitive test score at baseline, age (years), sex, intervention PREDIMED-Plus randomized groups, and participating center (south/north), education level (primary, secondary, or college), civil status (single, divorced or separated, married, widower), body mass index (kg/m^2^), physical activity (METs/min/day), smoking status (current, former, or never), energy-adjusted cumulative average alcohol consumption in g/day (and adding the quadratic term), depressive symptomatology (yes/no), diabetes prevalence (yes/no), hypertension prevalence (yes/no), hypercholesterolemia prevalence (yes/no), and energy-adjusted cumulative average consumption of food groups (vegetables, fruits, legumes, cereals, oils and fats, olive oils, biscuits, meat, fish, dairy, nuts [g/day], coffee, and tea [mL/day]). Significant values (*P* < 0.05) were highlighted in bold type

Figure 3 shows the sensitivity analyses conducted to check the robustness of the associations explored between energy-adjusted cumulative average dietary vitamin D intake and changes in cognitive function over the 2-year period after excluding individuals with baseline MMSE < 24 and extreme percentiles (< 2.5%, > 97.5%) of GCF *z*-score. After adjusting for potential confounders and treating energy-adjusted cumulative average dietary vitamin D intake as a continuous variable, the results remained consistent with the main analyses shown in Table 2 for global, general cognitive, and executive function and language when removing individuals with baseline MMSE < 24 and extreme percentiles (< 2.5%, > 97.5%) of GCF *z*-score (Fig. 3A, B and D, E; Table S4). Regarding executive function, the significant associations observed in Table 2 were lost in both analyses (Fig. 3C; Table S4). Comprehensive details regarding sensitivity analyses, particularly those treating energy-adjusted cumulative average dietary vitamin D intake as a categorical variable, are provided in Table S4.

## Discussion

The present study contributes to the limited body of longitudinal research investigating the prospective relationship between dietary vitamin D intake and cognitive function. It uniquely provides significant and novel findings over time in a population at high risk for cognitive decline, demonstrating positive associations with global cognitive function, executive function, and language domains, as well as a mitigated decline in the attention domain, even after adjusting for several potential confounders. Taken together, these findings underscore the importance of conducting clinical trials aimed at testing the effect of dietary vitamin D consumption on cognitive decline in older individuals vulnerable to cardiovascular diseases and cognitive impairment.

Cross-sectional studies examining the association between dietary vitamin D intake and cognitive function generally support a direct association, particularly in older individuals [14–17]. Conversely, in a cohort of women aged 80 years divided on the basis of inadequate or recommended weekly dietary vitamin D intake, a positive association between dietary vitamin D intake and cognitive function was observed only in those participants who met the recommendations [15]. Similarly, older adults over 60 years, grouped by inadequate or adequate daily dietary vitamin D intake, showed better cognitive function when their dietary vitamin D intake was adequate [14]. Additionally, in another study conducted in older individuals aged over 65 years with heart failure— a condition directly related to cognitive decline— each 1 µg/day increase in dietary vitamin D intake was reported to be cross-sectionally associated with an 8% higher cognitive performance, suggesting a dose–response effect [16]. Conversely, although no relationship between dietary vitamin D intake and cognitive function among frail older adults aged over 65 years has been previously reported, the authors attributed their findings to the low dietary vitamin D intake and a narrow range of intake within the study population [44].

Within the framework of our investigation, we observed that dietary vitamin D intake was associated with improvements in global cognitive function, executive function, and language, as well as less decline in the attention domain over a 2-year period. Of note, to help report the observed mean difference in age-equivalents for global cognitive function as a main outcome, the difference in changes in global cognitive function between the highest and the lowest group of cumulative average dietary vitamin D was roughly equivalent to 4.2 years of cognitive aging given consistent dietary vitamin D intake over time [45]. Regarding middle-aged cohorts, baseline dietary vitamin D intake, although positively associated with a limited subset of cognitive function domains both 5 years [19] and 13 years [18] thereafter, did not show a discernible relationship with any of the five cognitive function scores assessed in our study. Conversely, in relation to older populations, no prospective associations were reported between dietary vitamin D intake and cognitive function among men aged 70 years followed for 18 years [21], whereas within a cohort of women aged 80 years, higher baseline dietary vitamin D intake was associated with better cognitive function after 7 years of trajectories [20]. Although both investigations exclusively relied on the MMSE for cognitive assessment and solely measured dietary vitamin D intake at baseline, the cohort of males exhibited lower dietary vitamin D intakes, which may explain the lack of associations. The controversial results of the aforementioned studies may be attributed to methodological issues, including exclusive reliance on baseline dietary vitamin D exposure measurements, disparities between tools used for cognitive assessment [46], demographic heterogeneity of the studied population in relation to age, gender, geographic areas, the sample size, and the background of dietary vitamin D intake [47]. While the magnitude of the associations reported in our study may be considered of limited clinical relevance, it is important to note that our study consisted of older adults. Therefore, if such minimal differences can reveal a significant association, they may serve as a warning of cognitive impairment. Large-scale and well-designed longitudinal studies are warranted in the future to better elucidate a potential relationship between dietary vitamin D intake and cognitive function, especially in older adults at risk of vitamin D deficiency and cognitive decline [48].

The potential mechanisms underlying the correlations between dietary vitamin D intake and cognition have been the subject of proposed hypotheses. It is posited that lower dietary vitamin D intake may induce significant nitrosative stress within the brain, thereby potentially fostering cognitive decline among middle-aged and elderly individuals [49]. Adequate dietary vitamin D intake has also been reported to increase insulin sensitivity and reduce neuroinflammation in the brain, favoring cognitive function [50]. Given that the brain’s vitamin D receptor is involved in cognitive function, maintaining a dietary vitamin D intake near recommended levels could enhance bloodstream vitamin D concentrations and, consequently, confer cognitive benefits [51].

However, when assessing these associations, it is imperative to consider the potential for reverse causality, wherein cognitive impairments or diminished autonomy could precipitate suboptimal dietary habits [18]. Furthermore, despite the pervasive issue of insufficient dietary vitamin D intake, attributable in part to the restricted availability of dietary sources rich in vitamin D [52], principally found in fish products and to a lesser extent in eggs, oils, bakery, and dairy products [53], the elderly population confronts an augmented susceptibility to vitamin D deficiency. This scenario may be further compounded by several factors, including lower dietary vitamin D intake and heightened dietary recommendations [54], decreased outdoor activity, diminished synthesis of vitamin D in the skin, and impaired hepatic and renal function, which diminish the activation of vitamin D [44]. In conjunction with the aging process, individuals who are overweight, as observed in our study, may require higher dietary vitamin D intake to potentially preserve cognitive function compared to their younger or normal-weight counterparts [47]. Healthcare practitioners are encouraged to regularly inform elderly patients and their caregivers—particularly those who are overweight—about the possible benefits of maintaining adequate dietary vitamin D intake, conduct assessments for potential vitamin D deficiency in the bloodstream, and offer guidance to ensure sufficient dietary vitamin D consumption [48]. Consequently, intervention studies exploring the effect of increased dietary vitamin D intake could help to clarify its potential physiological benefits on cognitive function [55].

Our study presents notable strengths. First, its longitudinal prospective design facilitated the observation of temporal associations over a 2-year follow-up period, although this design does not establish potential causal relationships. Second, the comprehensive assessment of cognitive function utilized a diverse array of neuropsychological tests, enabling the measurement of composite scores across multiple cognitive domains. Third, the study benefitted from a large sample size, affording the adjustment of statistical models for various potential confounding factors. Lastly, the robustness of the findings was corroborated through the execution of different sensitivity analyses. Nevertheless, our study findings should be interpreted in light of certain limitations. Firstly, the potential for reverse causality and residual confounding persists, particularly from unmeasured factors not accounted for in the analyses. Secondly, the generalizability of the results may be limited to older populations with overweight/obesity and metabolic syndrome, precluding extrapolation to other populations. Thirdly, the absence of data pertaining to blood 25(OH)D levels, kidney function, sun exposure, seasonality, and racial background represents a notable information gap. Fourthly, as PREDIMED-Plus is a randomized controlled trial, though all the analyses were adjusted for the intervention group, the lifestyle advice that participants received could be affecting our findings [31]. Of note, dietary patterns and physical activity have a direct impact on body weight that is also critical, as overweight or obesity are linked to cognitive decline [56]. Furthermore, although we employed the energy-adjusted cumulative average of dietary vitamin D intake using the residual method to better represent long-term intake and reduce within-person variation and measurement error, reliance on a FFQ to estimate dietary vitamin D intake introduces potential sources of measurement error and recall bias, particularly given its dependence on participants’ memory and susceptibility to cognitive decline.

## Conclusion

In conclusion, our results suggest that a higher dietary vitamin D intake is associated with modest, favorable changes in cognitive function and may help to mitigate cognitive decline in the short term in older adults not following vitamin D supplementation with overweight/obesity and metabolic syndrome. Further research in this area aimed at increasing dietary vitamin D intake is warranted, especially in individuals at risk of vitamin D deficiency and cognitive impairment, given the rapidly expanding elderly population and the absence of curative treatment for cognitive decline.

## Supplementary Information

Below is the link to the electronic supplementary material.

Supplementary file1 (DOCX 121 KB)

## Data Availability

Data described in the manuscript, codebook, and analytic code will be made available upon request pending application and approval of the PREDIMED-Plus Steering Committee. There are restrictions on the availability of data for the PREDIMED-Plus trial due to the signed consent agreements around data sharing, which only allow access to external researchers for studies following the project purposes. Requestors wishing to access the PREDIMED-Plus trial data used in this study can make a request to the PREDIMED-Plus trial Steering Committee chair: jordi.salas@urv.cat. The request will then be passed to members of the PREDIMED-Plus Steering Committee for deliberation.
