# Supplementary material for: Dietary vitamin D intake and 2-year changes in cognitive function in older adults with overweight or obesity and metabolic syndrome
Source: GeroScience. 2025 May 6;47(5):6139–56. doi: 10.1007/s11357-025-01670-1 (PMC12634950; doi:10.1007/s11357-025-01670-1)
Supplement: Supplementary file 1 — Supplementary file1 (DOCX 121 KB) [file 11357_2025_1670_MOESM1_ESM.docx]

| **Table S1.-** Composite cognitive assessment equations^1^ | |
| --- | --- |
| **Composite cognitive domain** | **Composite component score** |
| *Global Cognitive Function^2^* | = $\frac{zMMSE + zCDT+ zVFT-a + zVFT-p + \left( -zTMT-A \right)+ \left( -zTMT-B \right)+ zDST-f+ zDST-b}{8}$ |
| *General Cognitive Function^3^* | = $\frac{zMMSE + zCDT}{2}$ |
| *Executive Function^4^* | = $\frac{zVFT-a + zVFT-p + \left( -zTMT-B \right) + zDST-b}{4}$ |
| *Attention^5^* | = $\frac{\left( -zTMT-A \right)+ zDST-f}{2}$ |
| *Language^6^* | = $\frac{zVFT-a + zVFT-p}{2}$ |
| Abbreviations: CDT, Clock Drawing Test; DST-b, Digit Span test - backward; DST-f, Digit Span test - forward; MMSE, Mini-Mental State Examination; TMT-A, Trail Making Test Part A; TMT-B, Trail Making Test Part B; VFT-a, Verbal Fluency tasks semantical; VFT-p, Verbal Fluency tasks phonological.  ^1^Standardized scores of the TMT-A and TMT-B were inverted, so that higher scores would represent better cognitive function.  ^2^The global cognitive function was determined by computing the mean standardized individual scores of all neuropsychological tests.  ^3^The general cognitive function composite included the MMSE score and the CDT score.  ^4^The executive function composite included the mean standardized individual scores of the VFT-a score, VFT-p score, TMT-B score, and DST-b score.  ^5^The attention composite included the mean standardized individual scores of the TMT-A score and the DST-f score.  ^6^ The language composite included the mean standardized individual scores of the VFT-a score and the VFT-p score. | |

| **Table S2.-**Baseline characteristics of the PREDIMED-Plus participants of dietary variables in overall and by quartiles of energy-adjusted cumulative average dietary vitamin D intake | | | | | | |
| --- | --- | --- | --- | --- | --- | --- |
|  |  | Categories of dietary vitamin D intake | | | |  |
|  | **Total** | **1^st^ quartile** | **2^nd^ quartile** | **3^rd^ quartile** | **4^th^ quartile** | **P-value^1^** |
| Total energy intake (kcal/day) | 2378 ± 553 | 2400 ± 580 | 2331 ± 552 | 2368 ± 554 | 2414 ± 520 | **0.001** |
| **Nutrients** |  |  |  |  |  |  |
| Carbohydrates (%) | 40.5 ± 6.8 | 41.7 ± 6.8 | 40.7 ± 6.9 | 40.5 ± 6.9 | 39.1 ± 6.3 | **0.001** |
| Protein (%) | 16.7 ± 2.8 | 15.7 ± 2.7 | 16.4 ± 2.7 | 17.0 ± 2.7 | 17.5 ± 2.8 | **0.001** |
| Total fat (%) | 39.5 ± 6.5 | 38.8 ± 6.6 | 39.5 ± 6.5 | 39.3 ± 6.6 | 40.2 ± 6.3 | **0.001** |
| Fiber (g/day) | 26.0 ± 8.7 | 24.5 ± 8.4 | 25.1 ± 8.3 | 26.4 ± 8.8 | 27.9 ± 8.8 | **0.001** |
| **Food groups** |  |  |  |  |  |  |
| Vegetables (g/day) | 324.9 ± 138.7 | 290.5 ± 129.9 | 305.8 ± 128.8 | 335.5 ± 140.1 | 368.0 ± 142.9 | **0.001** |
| Fruits (g/day) | 356.6 ± 205.1 | 336.3 ± 203.8 | 350.6 ± 196.5 | 364.6 ± 212.3 | 374.9 ± 205.6 | **0.001** |
| Legumes (g/day) | 20.7 ± 11.2 | 19.4 ± 10.3 | 20.1 ± 10.7 | 21.2 ± 11.7 | 22.1 ± 11.8 | **0.001** |
| Cereals (g/day) | 151.5 ± 79.3 | 158.9 ± 87.3 | 149.5 ± 78.7 | 151.2 ± 77.7 | 146.4 ± 72.0 | **0.001** |
| Total meat (g/day) | 148.5 ± 58.8 | 147.5 ± 59.4 | 146.8 ± 58.1 | 150.1 ± 58.8 | 149.6 ± 59.2 | 0.381 |
| Total fish (g/day) | 102.1 ± 47.2 | 71.9 ± 35.2 | 91.1 ± 38.2 | 109.2 ± 44.1 | 136.1 ± 45.2 | **0.001** |
| Nuts (g/day) | 6.0 ± 3.4 | 3.4 ± 1.5 | 5.0 ± 2.2 | 6.5 ± 3.0 | 9.3 ± 3.3 | **0.001** |
| Total dairy (g/day) | 341.0 ± 199.7 | 351.7 ± 207.7 | 336.3 ± 203.4 | 338.9 ± 189.2 | 337.1 ± 197.7 | 0.148 |
| Oils and fats (g/day) | 42.9 ± 17.3 | 43.3 ± 18.0 | 42.5 ± 17.0 | 41.9 ± 16.9 | 43.9 ± 17.3 | **0.014** |
| Olive oils (g/day) | 40.0 ± 17.0 | 39.8 ± 17.9 | 39.4 ± 16.6 | 39.2 ± 16.6 | 41.6 ± 16.9 | **0.001** |
| Biscuits (g/day) | 26.9 ± 30.1 | 30.7 ± 32.8 | 27.3 ± 29.7 | 25.9 ± 29.3 | 23.9 ± 28.0 | **0.001** |
| Coffee and tea (mL/day) | 88.8 ± 60.4 | 89.8 ± 61.0 | 87.8 ± 58.4 | 87.4 ± 60.4 | 90.4 ± 61.6 | 0.491 |
| Total alcohol (g/day) | 12.0 ± 15.6 | 13.4 ± 17.1 | 11.7 ± 15.6 | 11.4 ± 15.0 | 11.4 ± 14.4 | **0.001** |
| Data are presented as mean ± SD. Significant values (*p*<0.05) were highlighted in bold type.  ^1^P value for differences between categories of energy-adjusted cumulative average dietary vitamin D intake was calculated by one-way ANOVA. | | | | | | |

| **Table S3.-** Changes in cognitive function over 2 years of follow-up in overall and by quartiles of energy-adjusted cumulative average dietary vitamin D intake in the PREDIMED-Plus cohort. | | | | | |
| --- | --- | --- | --- | --- | --- |
|  | All | Categories of dietary vitamin D intake | | | |
|  |  | 1^st^ quartile | 2^nd^ quartile | 3^rd^ quartile | 4^th^ quartile |
| **Δ Global Cognitive Function (n)** | (n = 3737) | (n = 935) | (n = 934) | (n = 934) | (n = 934) |
| Mean ± SD dietary vitamin D intake | 6.54 ± 2.42 | 3.61 ± 0.82 | 5.48 ± 0.50 | 7.28 ± 0.56 | 9.79 ± 1.21 |
| Mean Δ [min,max] | 0.06 [-2.34,2.90] | 0.04 [-2.34,2.24] | 0.05 [-1.83,2.90] | 0.06 [-2.18,2.54] | 0.06 [-1.97,2.72] |
| **Δ General Cognitive Function (n)** | (n = 4536) | (n = 1134) | (n = 1134) | (n = 1134) | (n = 1134) |
| Mean ± SD dietary vitamin D intake | 6.56 ± 2.42 | 3.63 ± 0.82 | 5.51 ± 0.49 | 7.31 ± 6.40 | 9.81 ± 1.23 |
| Mean Δ [min,max] | 0.11 [-3.80,5.72] | 0.13 [-3.39,4.63] | 0.14 [-3.80,5.72] | 0.10 [-3.74,4.07] | 0.08 [-3.36,3.96] |
| **Δ Executive Function (n)** | (n = 3863) | (n = 966) | (n = 966) | (n = 966) | (n = 965) |
| Mean ± SD dietary vitamin D intake | 6.55 ± 2.42 | 3.63 ± 0.82 | 5.50 ± 0.49 | 7.28 ± 0.56 | 9.79 ± 1.21 |
| Mean Δ [min,max] | 0.06 [-2.51,2.80] | 0.05 [-1.87,2.27] | 0.03 [-2.48,2.80] | 0.08 [-2.49,2.61] | 0.07 [-2.51,2.00] |
| **Δ Attention** **(n)** | (n = 3926) | (n = 982) | (n = 981) | (n = 982) | (n = 981) |
| Mean ± SD dietary vitamin D intake | 6.56 ± 2.43 | 3.63 ± 0.82 | 5.51 ± 0.49 | 7.30 ± 0.56 | 9.81 ± 1.22 |
| Mean Δ [min,max] | -0.03 [-5.26,6.09] | -0.06 [-3.21,5.29] | 0.00 [-3.00,6.00] | -0.05 [-5.26,5.78] | -0.01 [-3.53,6.10] |
| **Δ Language (n)** | (n = 4657) | (n = 1165) | (n = 1164) | (n = 1164) | (n = 1164) |
| Mean ± SD dietary vitamin D intake | 6.58 ± 2.43 | 3.64 ± 0.83 | 5.53 ± 0.49 | 7.31 ± 0.56 | 9.82 ± 1.23 |
| Mean Δ [min,max] | 0.10 [-3.36,6.30] | 0.07 [-3.36,2.75] | 0.08 [-2.69,6.30] | 0.14 [-3.21,3.20] | 0.12 [-2.68,3.07] |
| Abbreviations: max, maximum; min, minimum; SD, standard deviation.  Data are presented as mean ± SD energy-adjusted cumulative average dietary vitamin D, and changes (Δ) [min,max] in cognitive function variables, respectively. | | | | | |

| **Table S4.-** Sensitivity analyses for the longitudinal association between energy-adjusted cumulative average dietary vitamin D intake and changes in cognitive function over a 2-year of follow-up in the PREDIMED-Plus cohort. | | | | | | | |
| --- | --- | --- | --- | --- | --- | --- | --- |
| ***Removal of participants with baseline MMSE <24 (n = 202)*** | | | | | | | |
|  | Continuous | | Categories of dietary vitamin D intake | | | |  |
|  | Dietary vitamin D intake (µg/day) | | 1^st^ quartile | 2^nd^ quartile | 3^rd^ quartile | 4^th^ quartile |  |
|  | **β [95% CI]^1^** | **p-value** | **β [95% CI]^1^** | **β [95% CI]^1^** | **β [95% CI]^1^** | **β [95% CI]^1^** | **P-trend** |
| **Global Cognitive Function (n)** | (n = 3653) | **–** | (n = 914) | (n = 913) | (n = 913) | (n = 913) | **–** |
| Mean ± SD dietary vitamin D intake | 6.54 ± 2.43 | **–** | 3.61 ± 0.81 | 5.49 ± 0.50 | 7.29 ± 0.56 | 9.79 ± 1.21 | **–** |
| Basic model | **10.1 [0.31,17.0]** | **0.004** | Reference | 0.19 [-4.79,5.17] | 3.19 [-1.79,8.17] | **6.46 [1.59,11.3]** | **0.004** |
| Multivariable-adjusted model | **11.5 [0.15,21.5]** | **0.023** | Reference | 0.94 [-4.15,6.03] | 4.04 [-1.55,9.64] | **7.30 [0.77,13.8]** | **0.018** |
| **General Cognitive Function (n)** | (n = 4420) | **–** | (n = 1105) | (n = 1105) | (n = 1105) | (n = 1105) | **–** |
| Mean ± SD dietary vitamin D intake | 6.56 ± 2.42 | **–** | 3.62 ± 0.82 | 5.51 ± 0.49 | 7.31 ± 0.56 | 9.81 ± 1.23 | **–** |
| Basic model | 0.31 [-0.63,1.26] | 0.514 | Reference | -0.93 [-7.50,5.64] | -1.62 [-8.07,4.82] | 1.92 [-4.62,8.46] | 0.585 |
| Multivariable-adjusted model | 0.04 [-1.31,1.39] | 0.953 | Reference | -1.33 [-8.16,5.49] | -2.19 [-9.45,5.07] | -0.49 [-9.22,8.22] | 0.901 |
| **Executive Function (n)** | (n = 3705) | **–** | (n = 927) | (n = 926) | (n = 926) | (n = 926) | **–** |
| Mean ± SD dietary vitamin D intake | 6.55 ± 2.42 | **–** | 3.61 ± 0.81 | 5.50 ± 0.50 | 7.29 ± 0.56 | 9.79 ± 1.21 | **–** |
| Basic model | **1.31 [0.56,2.07]** | **0.001** | Reference | -1.88 [-7.14,3.37] | 4.23 [-1.08,9.53] | **7.14 [1.89,12.4]** | **0.001** |
| Multivariable-adjusted model | 1.04 [-0.07,2.14] | 0.066 | Reference | -1.71 [-7.08,3.65] | 3.18 [-2.85,9.22] | 4.98 [-2.13,12.1] | 0.082 |
| **Attention** **(n)** | (n = 3759) | **–** | (n = 940) | (n = 940) | (n = 940) | (n = 940) | **–** |
| Mean ± SD dietary vitamin D intake | 6.56 ± 2.43 | **–** | 3.61 ± 0.81 | 5.50 ± 0.51 | 7.31 ± 0.56 | 9.81 ± 1.21 | **–** |
| Basic model | **1.07 [0.19,1.95]** | **0.017** | Reference | 4.17 [-2.00,10.3] | 2.37 [-4.20,8.95] | **9.14 [2.94,15.4]** | **0.009** |
| Multivariable-adjusted model | 1.04 [-0.20,2.28] | 0.100 | Reference | 4.49 [-1.74,10.7] | 3.43 [-3.85,10.7] | **9.48 [1.33,17.6]** | **0.040** |
| **Language (n)** | (n = 4454) | **–** | (n = 1114) | (n = 1113) | (n = 1114) | (n = 1113) | **–** |
| Mean ± SD dietary vitamin D intake | 6.57 ± 2.43 | **–** | 3.62 ± 0.82 | 5.52 ± 0.49 | 7.32 ± 0.56 | 9.81 ± 1.22 | **–** |
| Basic model | **2.02 [1.19,2.85]** | **0.001** | Reference | 1.19 [-4.64,7.02] | **7.34 [1.61,13.1]** | **11.9 [6.22,17.7]** | **0.001** |
| Multivariable-adjusted model | **1.56 [0.37,2.75]** | **0.010** | Reference | 0.53 [-5.41,6.47] | 5.16 [-1.29,11.6] | **7.91 [0.02,15.6]** | **0.023** |
| ***Removal of participants with extreme percentiles global cognitive function z-score at baseline (<2.5%, >97.5%) (n = 186)*** | | | | | | | |
|  | Continuous | | Categories of dietary vitamin D intake | | | |  |
|  | Dietary vitamin D intake (µg/day) | | 1^st^ quartile | 2^nd^ quartile | 3^rd^ quartile | 4^th^ quartile |  |
|  | **β [95% CI]^1^** | **p-value** | **β [95% CI]^1^** | **β [95% CI]^1^** | **β [95% CI]^1^** | **β [95% CI]^1^** | **P-trend** |
| **Global Cognitive Function (n)** | (n = 3551) | **–** | (n = 888) | (n = 888) | (n = 888) | (n = 887) | **–** |
| Mean ± SD dietary vitamin D intake | 6.54 ± 2.42 | **–** | 3.61 ± 0.81 | 5.49 ± 0.50 | 7.29 ± 0.56 | 9.79 ± 1.21 | **–** |
| Basic model | **1.04 [0.34,1.74]** | **0.004** | Reference | 0.67 [-4.42,5.76] | 3.06 [-2.02,8.14] | **6.72 [1.83,11.6]** | **0.004** |
| Multivariable-adjusted model | **1.13 [0.12,2.14]** | **0.028** | Reference | 1.23 [-3.94,6.40] | 3.70 [-2.00,9.41] | **7.02 [0.46,13.6]** | **0.027** |
| **General Cognitive Function (n)** | (n = 4350) | **–** | (n = 1088) | (n = 1087) | (n = 1088) | (n = 1087) | **–** |
| Mean ± SD dietary vitamin D intake | 6.57 ± 2.43 | **–** | 3.63 ± 0.81 | 5.51 ± 0.49 | 7.31 ± 0.57 | 9.81 ± 1.23 | **–** |
| Basic model | 0.39 [-0.57,1.34] | 0.428 | Reference | -0.57 [-7.30,6.14] | -1.02 [-7.61,5.57] | 2.02 [-4.60,8.63] | 0.563 |
| Multivariable-adjusted model | -0.16 [-1.54,1.22] | 0.819 | Reference | -1.69 [-8.63,5.25] | -2.83 [-10.2,4.56] | -2.22 [-11.1,6.65] | 0.614 |
| **Executive Function (n)** | (n = 3667) | **–** | (n = 920) | (n = 919) | (n = 919) | (n = 919) | **–** |
| Mean ± SD dietary vitamin D intake | 6.56 ± 2.42 | **–** | 3.63 ± 0.80 | 5.51 ± 0.50 | 7.29 ± 0.57 | 9.79 ± 1.22 | **–** |
| Basic model | **1.39 [0.63,2.15]** | **0.001** | Reference | -1.67 [-6.97,3.62] | 3.77 [-1.56,9.10] | **7.59 [2.36,12.8]** | **0.001** |
| Multivariable-adjusted model | 1.11 [-0.01,2.23] | 0.051 | Reference | -1.84 [-7.21,3.53] | 2.67 [-3.39,8.73] | 5.06 [-2.02,12.2] | 0.082 |
| **Attention** **(n)** | (n = 3740) | **–** | (n = 935) | (n = 935) | (n = 935) | (n = 935) | **–** |
| Mean ± SD dietary vitamin D intake | 6.57 ± 2.42 | **–** | 3.64 ± 0.81 | 5.51 ± 0.50 | 7.31 ± 0.56 | 9.81 ± 1.22 | **–** |
| Basic model | **1.06 [0.19,1.94]** | **0.017** | Reference | 5.88 [-0.24,12.0] | 1.77 [-4.81,8.36] | **10.3 [4.20,16.3]** | **0.005** |
| Multivariable-adjusted model | 0.94 [-0.29,2.17] | 0.134 | Reference | 6.13 [-0.07,12.3] | 2.81 [-4.46,10.1] | **10.4 [2.45,18.3]** | **0.032** |
| **Language (n)** | (n = 4471) | **–** | (n = 1118) | (n = 1117) | (n = 1117) | (n = 1117) | **–** |
| Mean ± SD dietary vitamin D intake | 6.58 ± 2.42 | **–** | 3.65 ± 0.82 | 5.54 ± 0.49 | 7.32 ± 0.57 | 9.82 ± 1.23 | **–** |
| Basic model | **2.03 [1.20,2.86]** | **0.001** | Reference | 1.30 [-4.52,7.10] | **7.57 [1.86,13.3]** | **11.8 [6.07,17.5]** | **0.001** |
| Multivariable-adjusted model | **1.41 [0.20,2.61]** | **0.022** | Reference | 0.08 [-5.83,5.99] | 4.66 [-1.81,11.1] | 6.61 [-10.7,14.3] | 0.052 |
| Abbreviations: CI, confidence interval. Basic models were adjusted for respective cognitive test score at baseline, age (years), and sex. Multivariable-adjusted models were further adjusted for intervention group, geographic area of the participating centers (south/north), education level (primary, secondary, or college), civil status (single, divorced or separated, married, widower), body mass index (kg/m^2^), physical activity (METs/min/day), smoking status (current, former, or never), energy-adjusted cumulative average of alcohol consumption in g/day (and adding the quadratic term), depressive symptomatology (yes/no), diabetes prevalence (yes/no), hypertension prevalence (yes/no), hypercholesterolemia prevalence (yes/no), and energy-adjusted cumulative average consumption of food groups (vegetables, fruits, legumes, cereals, oils and fats, olive oils, biscuits, meat, fish, dairy, nuts [g/day], coffee and tea [mL/day]). β-coefficients were estimated using linear regression models with robust standard errors to account for intracluster correlations. Linear trend was calculated by assigning the median values to each quartile of energy-adjusted cumulative average dietary vitamin D intake and treating these values across groups as a continuous variable in the linear regression models. Significant values (*p*<0.05) were highlighted in bold type.  ^1^β [95% CI] values are expressed as multiples of 10^-2^ (x10^-2^). | | | | | | | |
